# Modifying a multidisciplinary method to address challenging behavior in nursing home residents with dementia to involve family caregivers

**DOI:** 10.3389/frdem.2024.1444815

**Published:** 2024-09-30

**Authors:** Petra E. M. Tasseron-Dries, Hanneke J. A. Smaling, Jenny T. van der Steen, Wilco P. Achterberg

**Affiliations:** ^1^Department of Public Health and Primary Care, Leiden University Medical Center, Leiden, Netherlands; ^2^Medical Department of Stichting Warande (Nursing Home Organization), Zeist, Netherlands; ^3^University Network for the Care Sector Zuid-Holland, Leiden University, Medical Center, Leiden, Netherlands; ^4^Department of Primary and Community Care, Radboudumc Alzheimer Center, Radboud university medical center, Nijmegen, Netherlands

**Keywords:** dementia, nursing home, challenging behavior, pain, family involvement, intervention

## Abstract

**Introduction:**

Challenging behavior and pain are common in nursing home residents with dementia. Challenging behavior and pain can be related and are stressful for residents, family caregivers and healthcare professionals. The STA OP! method provides a step-by-step protocol to manage challenging behavior and pain in nursing home residents with dementia. However, this method does not include a prominent and active role for family caregivers.

**Methods:**

The STA OP! method was modified to include a role for family caregivers, in co-creation with family caregivers and healthcare professionals using elements of a realist approach. In separate meetings, two advisory groups comprised of family caregivers and professionals discussed ideas on how to involve family caregivers in STA OP!. Furthermore, barriers to involving family and possible solutions to overcome those barriers were discussed. Experts who had experience with the STA OP! method assessed the feasibility of the ideas in a nominal group technique meeting.

**Results:**

Thirty-eight ideas emerged in the advisory groups. The two ideas that generated the most discussion were Inviting family for a multidisciplinary meeting, and Assessment of pain in collaboration with family caregivers. Eventually, 21 ideas and suggestions to overcome possible barriers were included in a manual for the training of healthcare professionals in the adapted method, now called STA OP! *with family*.

**Conclusion:**

Healthcare professionals and family caregivers collaborated well to shape the involvement of family caregivers in this method for managing challenging behavior and pain. The collected ideas supported by all involved resulted in a modified method: STA OP! *with family* and can now be tested in daily practice.

## Introduction

Behavior that challenges (challenging behaviors) such as apathy, depression, and aggression are common in people with dementia living in a nursing home (Van Der Linde et al., 2016; Zhao et al., 2016). More than 80% of nursing home residents with dementia experience some neuropsychiatric symptoms at some point (Selbæk et al., 2013; Zuidema et al., 2007). Challenging behavior often stems from unmet psychological, social, or physical needs (Kovach et al., 2005; Cohen-Mansfield, 2000). Pain, a physical need, is common among nursing home residents, and can be an underlying cause for challenging behavior (Achterberg et al., 2021; Husebo et al., 2016; Kovach et al., 2005). Despite the development of pain observation scales, pain in dementia is still poorly recognized and undertreated (Achterberg et al., 2021; Tait and Chibnall, 2008).

Challenging behavior has a major negative impact on all persons involved, including formal and informal caregivers (Selbaek et al., 2014). It affects residents' quality of life and is a burden for their family caregivers (Chekani et al., 2021). It is therefore often a cause for admission to the nursing home (Nunez, 2021). However, caring for this resident is also stressful for nursing staff, in part because the behavior influences other residents (Klaver et al., 2021; Zwijsen et al., 2014).

It is widely acknowledged that family involvement in the care for nursing home residents with dementia, including preventing and reducing challenging behavior, is important and positively affects the resident's quality of life (Hovenga et al., 2022; Tasseron-Dries et al., 2023; Gaugler, 2005; Robison et al., 2007; Roberts and Ishler, 2017). Being involved can also decrease family's caregiving burden (Robison et al., 2007; Irving, 2015) and staff burnout (Robison et al., 2007). Family caregivers' unique knowledge of their relative's life can be useful in the management of challenging behavior and pain. However, there are still many barriers to involving family, and implementing this involvement in daily practice appears to be difficult (Tasseron-Dries et al., 2021; Hovenga et al., 2022). As a result, caregivers' understanding of behavior may be an underused resource in strategies to manage pain and challenging behavior.

A multidisciplinary intervention used in the Netherlands for the management of challenging behavior is the STA OP! method (Pieper et al., 2018). STA OP! is the Dutch version of the Serial Trial Intervention-protocol (STI) (Kovach et al., 2006b), a stepwise method to assess, manage, and monitor challenging behavior and pain in people with advanced dementia (Pieper et al., 2011). A study showed that the STA OP! method reduces challenging behavior in residents with advanced dementia (Pieper et al., 2016; Kovach et al., 2006a). Also, the STA OP! method improved the quality-of-life domains “restless tense behavior” and “social isolation” and decreased observed pain (Pieper et al., 2016; Klapwijk et al., 2018). Furthermore, the intervention increases healthcare professionals' awareness of pain signals of and challenging behaviors in residents (Pieper et al., 2018). However, the current intervention does not include an active and clear role for family caregivers. Involving family caregivers in the approach to management and prevent challenging behavior can contribute to partnership in care and may improve the intervention, also in terms of positive effects for family and healthcare professionals.

Co-creation can help develop successful, relevant and widely accepted interventions that improve quality of life by involving all stakeholders who share responsibility for developing and delivering a viable, high-quality intervention. Furthermore, co-creation helps adjust to the needs of all involved (Pearce et al., 2020). Including the input from different perspectives enhances adoption and implementation of the intervention.

The aim of this study is to modify the STA OP! intervention and give a prominent role to family caregivers in managing challenging behavior and pain in nursing home residents with dementia. A three-stage process is used and the adjusted intervention will be referred to as STA OP! *with family*.

## Materials and methods

### Intervention

The STI and STA OP! method is based on the theory of unmet needs (Cohen-Mansfield, 2000), which assumes unmet needs of the resident as an explanation for challenging behavior. We hypothesize that the method benefits healthcare professionals by offering a structured approach to the early detection and management of pain and challenging behaviors. Theoretically, this strategy enables caregivers to intervene faster and with more focus. Additionally, it is suggested that the approach promotes communication and collaboration among multidisciplinary professionals, which may improve meeting residents' needs. It is expected that the strategy will improve patient outcomes by enhancing these areas of care. STA OP! consists of the following steps and requires if a step is unsuccessful:

*Step 0: assessment of basic needs*

Does someone need to go to the toilet, is someone hungry, is someone uncomfortable? Healthcare professionals have an important role in first discovering the underlying need and then fulfilling the needs (Kovach et al., 2005).

*Step 1: pain and physical needs assessment*

Involves completing pain assessment and physical examination by the doctor/nurse practitioner. Is there a physical cause for the behavior, such as an infection or constipation?

*Step 2: affective needs assessment*

Map activities, environmental factors causing the behavior together with the psychologist.

*Step 3: trial non-pharmacological comfort interventions*

Massage, aromatherapy, music therapy, projecting soft colors on the wall.

*Step 4: trial analgesics*

Even if there is no certainty that pain is the cause, this (“blind” use of pain medication) is an essential step in the method.

*Step 5: trial psychotropic drugs or consultation*

Consultation of a psychiatrist, only after all other steps have been completed.

### Study design

The current study is part of the Cared-4 study,^1^ which explores the impact of the modified STA OP! *with family* on residents, family, and healthcare professionals using a pre-post design. This study aims to modify the STA OP! intervention in co-creation with family caregivers and healthcare professionals to include a more prominent role for family caregivers. The development of the STA OP! *with family* intervention is described using three iterative stages based on the realist approach (Pawson and Tilley, 1997) and the framework of Walshe et al. (2019). The realist approach maps how a complex social intervention works in practice and in what circumstances. The ACCORD (ACcurate Consensus Reporting Document) (Gattrell et al., 2022) checklist was used for reporting about the study.

**Figure 1:**
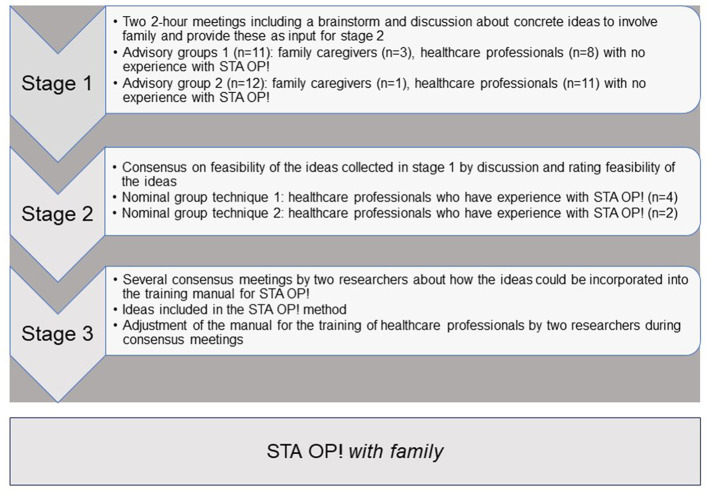
Data collection in the stages of the development of the STA OP! *with family*.

Co-creation provides all stakeholders the opportunity to influence the outcome (Pearce et al., 2020). Stakeholders were actively involved in advisory groups in stage 1 and during a nominal group technique meeting in stage 2 (NGT). Figure 1 shows an overview of the data collection in the three stages of the development of STA OP! *with family*. The advisory groups were completed in April 2022 3 weeks apart. The first NGT group was conducted in May and the second in August 2022.

### Recruitment of participants

Family caregivers and healthcare professionals were informed about the Cared-4 study, using the information channels (e.g., newsletters, intranet, email) of a nursing home in the center of the Netherlands. The inclusion criteria are specified in Table 1. For the advisory groups, a staff member of the center for consultation and expertise (CCE; an organization that supports healthcare professionals who need help resolving severe issues with residents with challenging behavior) was specifically invited because of their expertise with severely challenging behavior. For the NGT group, healthcare professionals who had either participated in a nursing home STA OP! pilot, or who worked in a nursing home that had recently implemented STA OP!, or who participated in the previous STA OP! study (Pieper et al., 2011) were asked to participate. Potential participants received an information letter and consent form. The researcher contacted the healthcare professionals who expressed an interest in participating in the study.

**Table 1:** Inclusion criteria for advisory groups and NGT (expert) group.

	**Advisory groups**	**NGT (expert) group**
18 years of age	X	X
Adequate use of Dutch language	X	X
Relatives (i.e., legal representative/ family caregiver of a resident with an indication for a psychogeriatric facility and diagnosed with dementia)	X	X
Healthcare professionals working on a psychogeriatric ward	X	X
Managers of a psychogeriatric ward	X	X
Members of the client council	X	X
Experience with STA OP! method		X

Thirteen people were invited to participate in the first advisory group and 15 for the second group. For the NGT meeting, nine healthcare professionals were initially invited to participate. Eventually, 11 and 12 persons respectively participated in the advisory groups, and 6 persons took part in the NGT meeting. All participants received a €20 gift card in appreciation for their participation.

### Data collection

*Stage one: advisory groups*

Stage one included two advisory groups with family caregivers and healthcare professionals who had no experience with STA OP!. The aim of an advisory group is to provide input and advice on interventions. Their focus is on testing ideas and generating useful recommendations (Koskinas et al., 2022). The aim of the advisory groups was to collect ideas for structurally involving family in the STA OP! intervention to address challenging behavior and pain in nursing home residents with dementia.

Two researchers (PT and HS) led the 2-hour advisory groups at the nursing home. The meeting started with a brief introduction about the aim of the session and the Cared-4 study. Next, background information about challenging behavior, the STA OP! method, and family involvement were presented to the group (15 min). Subsequently, participants brainstormed in pairs about concrete ideas to involve family caregivers in STA OP! The ideas were written down and shared with the group. Each idea was discussed, including any barriers and solutions for those barriers. Finally, each participant selected their top-5 best ideas. The ideas that received more than 3 votes were directly included as input for the NGT session. A summary of these ideas was sent to all participants after both advisory group sessions. A standard operating procedure was prepared in advance of the meetings describing the time and instructions per topic. One researcher led the sessions, while the other researcher monitored the balance between healthcare professionals and family, ensured all participants were able to provide their input, and was responsible for time management.

*Stage two: nominal group technique*

A 2-h nominal group session with six healthcare professionals who have experience with STA OP! was conducted in stage two. NGT promotes the development of diverse ideas through individual brainstorming and enables consensus building by prioritizing and discussing ideas (Mcmillan et al., 2016). The feasibility of the ideas, contributed by stakeholders, is assessed by participants who have experience with the method. All ideas considered important by stakeholders are given a chance, which prevents the unfounded discarding of unfeasible ideas and participants losing enthusiasm. The aim of the NGT session in this study was to reach consensus on the feasibility of the ideas collected in the advisory group(s) and decide which ideas needed to be included in the modified STA OP! *with family* intervention.

Prior to the meeting, the participants rated the feasibility of each idea to involve family caregivers in STA OP! using a 5-point Likert scale. The questionnaires were processed anonymously. A feasible idea must be generalizable and include a suggestion to involve family caregivers that all nursing homes can consider. The NGT meeting focused on reaching a general agreement on which interventions that aimed at an active role for family caregivers in the approach to challenging behavior and pain should be included in the modified STA OP! intervention.

Due to unforeseen last-minute circumstances, only 4 of the 6 participants were able to attend the NGT meeting. An additional meeting was scheduled with the two participants, which generally had the same format, with the two participants also being shown the discussion on the feasibility of the ideas from the first group.

The initial NGT meeting was led by two researchers (PT and JS), while a student assisted. The additional meeting was led by one researcher (PT). Both meetings were held online. The general feasibility ratings that were filled in prior to the meeting were discussed within the group. Finally, each participant rated the individual ideas as feasible or not feasible. After this rating, the ideas deemed feasible by 80% of the participants, were included in the different steps of the method.

*Stage three: developing the manual for the STA OP! with family method*

We adjusted the training manual of STA OP! for the healthcare professionals based on the input from the advisory groups and NGT meeting. Two researchers (PT and HS) discussed in several consensus meetings how the ideas for family involvement that were deemed feasible could be incorporated in the training manual for STA OP!. The ideas that were deemed feasible, were placed in the structured steps of the intervention. Next, the manual for the training was gradually updated and further developed by PT, WA, and JS. Similarly, the manual for the STA OP!-trainer was modified by PT together with a certified trainer of the STA OP! method.

### Data analysis

*Stage one: advisory groups*

The meetings of the advisory groups were audio-recorded and summarized. Similar ideas collected were merged by two researchers (PT and HS). Descriptive statistics were conducted with SPSS version 29 to describe participant characteristics.

*Stage two: nominal group technique*

“Poll Everywhere” (Polleverywhere, 2020) was used in the NGT meetings to collect the participants' individual votes and calculate percentages. Poll Everywhere can be used, for example, during a presentation to stimulate interaction and to get answers to particular questions. Participants' responses are administered in anonymously. The data comprise audio-recordings, summaries, and field notes. The feasibility ranking of the ideas to involve family caregivers in STA OP! was examined in the content analysis. Descriptive statistics conducted with SPSS version 29 were used to describe participant characteristics.

*Stage three: developing of the manual for the STA OP! with family method*

Two researchers (PT, HS) performed a content analysis (Graneheim et al., 2017) to identify themes from the meeting transcripts. This approach enables systematic identification of patterns, themes and meanings within complex, qualitative data and contributes to depth, reliability and validity of research results. The findings of the qualitative analysis were then discussed in the research team to guarantee analytical rigor.

## Results

In co-creation with 23 participants, a total of 21 ideas on how to involve family caregivers in the STA OP! method were incorporated in the modified STA OP! *with family* method. Table 2 presents a description of the participants.

**Table 2:** Characteristics of the participants of the advisory groups (*n* = 23) and NGT (expert) group (*n* = 6).

**Sociodemographic**	**Statistic**	**Advisory group**	**Nominal group**
Total number of participants	n	23	6
Healthcare professionals total	n	19	6
**Sex**			
Female	n	16	6
Male	n	3	0
Other	n	0	0
Age	Mean (SD)	43.3 (12.8)	48.8 (11.1)
	Min-max	22–61	33–63
**Profession**			
Manager	n	3	1
Physician/nurse practitioner	n	3	0
Nursing staff	n	7	1
Psychologist	n	1	2
Activity coordinator	n	2	0
CCE staff member	n	1	0
STA OP! trainer	n	0	1
Other multidisciplinary members	n	2	1
Family caregivers total	n	4	NA
**Sex**			
Female	n	2	NA
Male	n	2	NA
Other	n	0	NA
Age	Mean (SD)	65.8 (12.2)	NA
	Min–max	48–76	NA
**Relationship**			
Spouse	n	2	NA
Child	n	2	NA

CCE, center for consultation and expertise; NA, not applicable; NGT, nominal group technique; n, number of participants; Sd, standard deviation; min-max, minimum and maximum.

*Stage one: advisory groups*

We scheduled two advisory group meetings with a total of 23 participants, including 4 family caregivers, 2 wives and 2 sons. These meetings produced a total of 38 ideas. Twenty-two ideas emerged in the first group, which covered exchanging information with family caregivers (*n* = 7), communication (*n* = 7), observation of and reporting on the resident (*n* = 5) and meaningful activities (*n* = 3). Another 16 ideas were collected in the second group, relating to meaningful activities (*n* = 2), sharing knowledge about challenging behavior (*n* = 3), the resident's life course (*n* = 3), facilitating family to participate in daily life in the nursing home (*n* = 2), managing challenging behavior (*n* = 2), and communication with family (*n* = 4). Participants' rating of the top-5 best and most valuable ideas resulted in 7 selected ideas with more than 3 votes in both advisory groups. Interestingly, although only endorsed in the first group, 'involving family in pain observation and anamnesis' was discussed in both groups. A summary of these ideas is included in Supplementary material A.

Possible barriers to family involvement in STA OP! *with family* and suggestions for solutions were then discussed and identified by the advisory groups. An overview of these barriers and solutions is shown in Table 3.

“*My own experience, the relationship you had with the parent in the past, the closer you are to the parent, the easier it is to continue caring; and others who are further away or who based on the past think: this is where it stops... I think that that's also, how close you are to the parent, your own experiences and feelings.... Then as a nursing home you can put as much energy into it as you like, but I doubt it will get you very far.”—Family caregiver about barriers to family involvement*

**Table 3:** Potential barriers to family involvement in STA OP! *with family* and suggestions for improvement categorized by topic and randomly discussed in the advisory groups.

**Topic**	**Barriers**	**Suggested solutions**
Resident's life story	• Family is not always able to provide a comprehensive life history of the resident. • Unclear who is responsible for keeping resident information up-to-date and in the awareness of everyone involved in the resident's care	• A brief one-page summary of a life history is enough. • One month after admission to the nursing home, invite family to write down the resident's life and explain to them why this is important. • Keep the life history and other information up-to-date by having regular contact with family about the resident and their routines and interests. • The primary professional caregiver takes the lead in collecting and updating the resident's life story with input from family. • Create a mind map for the resident (combine pieces of information).
Sharing knowledge	• Insufficient family meeting attendance due to COVID-19 restrictions. • Too much repeating of information at family meetings, making it less interesting for family members who have attended before. • Family caregiver having less experience in interacting with people with dementia and experiencing emotional burden because of their family member's cognitive decline	• Discuss mutual expectation regarding roles and tasks of healthcare professionals and family in the care for the resident • Psychoeducation for family caregivers by physician or nursing staff • Professionals being attentive to the emotional pain of family caregiver
Activities for family to be involved in	• Some risk attached to activity in which family is involved (e.g., assisting resident with transfer using a device) and is therefore a concern. Limitations on what family can be involved in, for example based on nursing home policy • Personal circumstances of the family caregiver (e.g., age, burden, availability of transport) • No overnight accommodation capacity • Charging family caregiver for dinner or overnight accommodation • Healthcare professionals experience presence of family as intimidating ('looking over my shoulder') • Possible impact of family's approach on the resident (e.g., if the resident becomes agitated by their visit) • Conflicting scores in pain assessment by family and professionals	• Offer family caregivers education or organize a workshop by healthcare professionals • Give family a choice in what they want to be involved in • Provide guidance to family caregiver in dealing with challenging behavior, through informal contact and by listening to family caregiver when they visit • Awareness among professionals of the impact on family • Healthcare professionals provide psychoeducation to family caregivers • Professionals perceive family caregivers as part of the resident group • Ask family caregiver to create a photo collage of the things that are important to the resident so this information is readily available to the care professionals • Educate and/or explain how to assess pain to family caregivers
Relationship between resident, family caregiver and healthcare professionals	• Poor relationship between resident and family caregiver • Cultural differences (e.g., religion) between family caregiver and healthcare professional	• Ask family caregiver to fill in a questionnaire about their relationship with the resident • Discuss cultural differences and specials needs regarding those differences in a care plan meeting • Healthcare professionals being aware of the relationship between family caregiver and resident
Protocol	• Laws and regulations that limit family caregivers' involvement	• Be open to complex interventions that must comply with laws and regulations, such as coercive measures, where professionals collaborate with the family to decide whether the measure is proportional and the benefits outweigh the disadvantages
Communication	• Task-oriented clinical reporting by healthcare professionals that indicates nothing about the resident's mood • No follow-up on the special concerns indicated in the report by healthcare professionals	• Describe resident's mood and emotions (happy, sad) in the daily report so family caregiver can read how the resident is doing. • Follow-up regarding the special concerns mentioned in the daily resident reports, including actions/interventions taken
	• Nursing home vision of providing good care (how do we interact with residents with dementia) is unknown to family caregivers or unclear • Routines residents had in the home setting are no longer feasible in the nursing home setting, which offers different possibilities and challenges • Healthcare professionals not listening to the residents' and family caregivers' needs • Family not asked for input or invited to multidisciplinary meetings • No family present, resident having a legal representative	• The nursing home vision is already clear to family caregivers before the resident is admitted, e.g., included in a brochure or website • The vision of each nursing home should be: “it is the resident's home (and we work there)” • Organize gatherings for family caregivers to share experiences with other family caregivers • Flexibility in managing daily schedule on a ward in the nursing home • Discuss daily routines of the resident upon admission • Combine care plan meetings with multidisciplinary meetings to give family caregivers the opportunity to attend a multidisciplinary meeting

“Scheduling a multidisciplinary meeting that includes a family caregiver as a multidisciplinary member” was a plan that received considerable endorsement. On the other hand, this idea also generated the most discussion due to logistical problems that were considered a barrier, especially by the professionals.

“*It feels a bit forced that the care plan conversation is separate from the multidisciplinary team meeting. In my opinion, you could combine them. I can also see things in the behavior that are abnormal and that I would like to see discussed”—Family caregiver*

Having family conduct a pain assessment also created considerable discussion. Generally, family indicated that their lack of expertise in that area was a barrier.

“*I do think you need experience to do that, observation, I don't think it's something you just do” -Healthcare professional*

Additional ideas were added to the list for the NGT group, based on the premise that by excluding ideas based on individual preferences, valuable ideas could be lost. These ideas initially received insufficient votes to be included but during the discussion with the group and researchers (PT and HS) were marked as valuable enough to present to the experts in the NGT group. Ideas that were unsuitable because of insufficiently concrete description or that had received no votes were excluded. The researchers (PT and HS) adapted the 38 ideas from the advisory groups by combining similar ideas and formulating ideas more concretely. This resulted in 24 ideas being used as input for the NGT meeting.

Some of the barriers to family involvement discussed in the meetings were demanding family, staff not seeing family as partner in care, time restraints, and lack of time experienced by staff. Activities that match family caregiver and resident's needs and a change in culture where family are no longer seen as visitors, but as collaborative partners were mentioned as facilitating for family involvement in the STA OP! method. All participants agreed that a change in the organization's culture is needed to really involve family in the care for the resident and see them as partners.

“*You also need to make it possible in a practical sense. We now have a family that showers their mother almost every day. But in the beginning they also thought: “well where do you leave the towels and these items are not there...” so you also have to make sure that those items are in that room, that they know where to leave the dirty laundry. Practical things like that. An obstacle for the care staff was: oh...the family takes over the showering five days a week, but how will I still know how the client is doing and if she still lifts her arms properly and is that painful for her....so it does require us to coordinate...”—Healthcare professional*

Finally, participants reported that it was an inspiring and informative meeting. They indicated that the meeting could be extended because there is still much to consider and to discuss around family involvement.

“*The 'behavior meetings' we have with each other on the ward are very suitable to do that... to map out the situation, so to speak, and how do we then deal with that? How can we help each other?”—Healthcare professional about collaboration with family caregivers*

*Stage two: NGT*

The questionnaire used to rate the feasibility of each idea from the advisory groups to involve family caregivers in STA OP! resulted in seven ideas being scored (probably) not feasible (Table 4). All 24 ideas from the advisory groups, including the ideas that were questioned beforehand, were discussed with the group. Ideas deemed feasible by 80% of participants were adopted. However, during the discussion after the second individual vote, consensus was reached that, although not feasible in every nursing home, all ideas could be useful and feasible as suggestions within STA OP! *with family*. The ideas “inviting family caregivers to the multidisciplinary meeting”, “shaping the daily hands-on care together with family caregivers”, “where caregiver can help, observe, and provide suggestions”, and “facilitating overnight accommodations” were rated as feasible by 80% of the participants. The remaining ideas scored 100%.

**Table 4:** Ideas collected in the advisory groups that participants in the NGT meetings rated as less feasible prior to the NGT.

**Ideas from advisory groups**	**Reason (s)**	**Number of participants who rated the idea as less or not feasible (*n* = 6)**
Pain assessment scale completed by family caregivers	Family caregiver has no experience, staff have no time for instruction	3
Provide training on daily care to family caregivers and involve them in daily care (e.g., washing and dressing)	Difficult to schedule and intimacy can be a barrier	3
Facilitating (overnight) accommodation	No rooms available, too expensive	5
Video call with family caregiver at times of challenging behavior	Very burdensome for family caregivers	1
Call list with names of family caregivers who can be contacted in case of escalation of behavior (Later in the development process changed into: *Engage resident's social network)*	Very stressful, especially if only one family caregiver is available	1
Regular contact of staff that know resident best with family	Stressful and time consuming for already busy nursing staff	1
Including family caregiver as a member of the multi-disciplinary team and therefore present at regular multidisciplinary meetings	Difficult to schedule as other residents are also discussed during those meetings	1

Barriers to family involvement that were mentioned in both NGT meetings were scheduling issues, no family to involve, and assumptions nursing staff make about family caregivers that hinder optimal family involvement: 'it is best for family caregivers to engage in enjoyable activities instead of taking on all care responsibilities' and 'family perceive residents based on their prior identity rather than the person they are now'. Also, staff taking over all care was seen as a limitation to involve family in the STA OP! method.

“*Then the conversation was initiated with two sisters and they were asked: what things do you not want us take away from you? In the contact with your mother? And one sister said very firmly, she also started crying...: I took care of her for so long. I was a nurse. I live around the corner. I would very much like to, I live around the corner, like to do that care moment three times a week. Well, that was inconvenient for nursing staff... the other sister said: don't call me, I'm not going to do that.”—Healthcare professional in NGT meeting*

During the additional NGT meeting, both participants rated all ideas as feasible. It was decided to include all 24 ideas in the method, as the majority had judged them to be feasible, provided that family involvement is tailored, the ideas are used for inspiration, and staff and family can decide for themselves whether an idea is feasible.

Furthermore, the discussion from the first NGT meeting was reviewed and some ideas were refined by participants. For example: “*giving family caregivers the opportunity to attend the multidisciplinary meeting”* instead of making it a standard procedure or an obligation. Also, it was suggested that family could make a video for staff to show the resident at times of challenging behavior. This idea was considered as less burdensome for family caregivers than being called *ad hoc* to have the resident hear the family's voice as a means to reduce challenging behavior. Finally, asking family caregivers upon admission to the nursing home: “*What care should I not take away from you*?” was deemed a suitable starter for a conversation with family caregivers about family participation. In conclusion of the meeting, participants expressed their consent to the first group's classification of the ideas into the different steps.

*Stage three: development of STA OP! with family caregivers*

Researchers PT and HS refined the phrasing of the ideas based on the results of stages one and two, and combined similar ideas. This resulted in 22 ideas (Table 5) that were added to the STA OP! training manual. Two of the 24 feasible ideas were merged with another idea or excluded. The first idea, to video call family at times of challenging behavior, was considered to be very stressful for family. In addition, there was already an idea to have family members record a video in advance that could be shown to the resident at times of challenging behavior being present. The second idea—creating a list of contacts to call in case of challenging behavior—was integrated into the broader idea of involving the resident's social network, meaning working together to create a plan that involves the social network in managing and preventing challenging behavior. Each idea was added to one of the steps of the method or adopted as a general idea not related to a particular step. Some ideas were suitable for multiple steps or could be categorized in one of the steps in parallel to a general idea. The training manual was also updated, for example by adding new contemporary interventions such as animal robots. The manual for trainers of the present STA OP! was adjusted by the researcher (PT) in collaboration with a teacher of the present STA OP! method. The modified manual for the multidisciplinary teams included a chapter on communication and collaboration with family caregivers, which takes into account the 4 roles family caregivers have in caring for their relative, namely: expert (i.e., know best what the resident needs), collaborative partner, personal relationship of the resident, and care recipient (Twigg and Atkin, 1994).

**Table 5:** The 22 ideas to involve family caregivers mapped as general idea or to one of the steps in STA OP!, including the agreement on feasibility.

	**Idea mapped as general idea or in step 0 to 5 in STA OP!**	**Agreement on feasibility**
*General idea*	Give family caregivers the opportunity to attend a multidisciplinary meeting as a member of the multidisciplinary team.	*80%*
	Get to know the family caregiver; for example, by asking them about their relationship with the resident and their life before admission to the nursing home.	*100%*
	Staff and family caregivers share their feelings regarding the challenging behavior caregivers (e.g., in the regular meetings that nursing staff has with the psychologist). This improves mutual understanding, which also enables professionals to provide better guidance and knowledge to family.	*100%*
	Professional caregivers who are most involved in the resident's care have regular in-person meetings with the family caregiver.	*100%*
	Organize regular family meetings in which professionals and family caregivers have the opportunity to share experiences and knowledge.	*100%*
	Besides medical and nursing matters, also report on the mood or emotional state of the resident and how things are really going in the resident's patient file. E.g., not “she went to toilet”, but “she felt very happy, laughed all the time and sang along with ….”	*100%*
*Step 0* *Assessment basic needs*	Provide psychoeducation (information) to family members about the course of dementia. Also, pay more attention to knowledge transfer in informal contact, specifically about the causes of and dealing with challenging behavior and pain.	*100%*
	Discuss the challenging behavior with family caregivers in an informal conversation, multidisciplinary consultation, or care plan meeting. When appropriate, support family caregiver by providing information about dementia and challenging behavior.	*100%*
	Engage the resident's social network in the approach to prevent and manage challenging behavior, for example, by jointly making a plan to involve them. Do not just focus on the primary caregiver.	*100%*
	Shape the daily hands-on care together with family caregivers; where can the family caregiver help, observe, and make suggestions.	*80%*
	Discuss daily routines, behavior, and personality of resident with family caregivers to serve as input for a person-centered care plan.	*100%*
	Provide a training in daily care for family caregivers.	*100%*
*Step 1* *Pain and physical needs assessment*	Give family caregivers the opportunity to attend a multidisciplinary meeting as a member of the multidisciplinary team.	*80%*
	Have family caregiver complete a pain assessment scale.	*100%*
	Complete a pain assessment together with family caregiver.	*100%*
*Step 2* *Affective needs assessment*	Facilitate resident and family caregiver eating together.	*100%*
	Provide psychoeducation (information) to family members about the course of dementia. Also, pay more attention to knowledge transfer in informal contact, specifically about the causes of and dealing with challenging behavior and pain.	*100%*
	Besides medical and nursing matters, also report on the mood or emotional state of the resident and how things are really going in the resident's patient file. E.g., not “she went to toilet”, but “she felt very happy, laughed all the time and sang along with ….”	*100%*
	Map the resident's life story together with family caregivers, using a mind map or collage. Put up a notice board in the resident's room displaying their hobbies, leisure activities, taste in music, and eating and sleeping habits.	*100%*
	Relatives and staff consider appropriate activities for family caregivers to do together with the resident or for the resident (e.g., go for a walk, tidying up the closet, eating together).	*100%*
	Staff and family caregivers share their feelings regarding the challenging behavior caregivers (e.g., in the regular meetings that nursing staff has with the psychologist). This improves mutual understanding, which also enables professionals to provide better guidance and knowledge to family.	*100%*
	Provide clarity to family caregivers on what activities in the resident's care they can participate in. Support them with concrete examples based on their personal needs regarding their involvement in the care of their relative.	*100%*
	Discuss daily routines, behavior, and personality of resident with family caregivers to serve as input for a person-centered care plan.	*100%*
	Create a photo book of the resident's life with pictures of their children, grandchildren, and other important relatives and friends.	*100%*
	Make a video of the family caregiver to show to the resident at times of (severe) challenging behavior.	*100%*
*Step 3* *Trial non-pharmacological comfort interventions*	Facilitate overnight accommodation for family caregivers.	*80%*
	Provide a training in daily care for family caregivers.	*100%*
*Step 4* *Trial analgesics*	Have family caregiver complete a pain assessment scale.	*100%*
*Step 5* *Trial psychotropic drugs or consultation*	Provide psychoeducation (information) to family members about the course of dementia. Also, pay more attention to knowledge transfer in informal contact, specifically about the causes of and dealing with challenging behavior and pain.	*100%*

## Discussion

This study shows that it is possible to adapt a multidisciplinary method for dealing with challenging behavior and pain to incorporate a role for family caregivers in a systematic way. With the help of family caregivers and professionals, a wealth of ideas was collected that we—as a research group—might not have thought of beforehand. We learned that these ideas, rather than a change in the structure of the intervention, mainly concerned concrete and practical ideas to involve family caregivers.

Using three of the four stages from the framework of Walshe et al. (2019) supported the process of developing the STA OP! *with family* together with family caregivers and professionals. A variety of ideas were collected and rated on feasibility in daily practice during three sessions of co-creation with family caregivers. This structured process of collecting ideas on how family caregivers can be involved in STA OP! ultimately led to 21 ideas and a tailored training and training manual for the multidisciplinary teams. Modification of this multidisciplinary method was accomplished in a meaningful and structured way, in good collaboration with family caregivers.

Despite the different perspectives of professionals and family caregivers, with proper consultation and collaboration family caregivers are willing to be and stay involved in the care for their relative, as other research already highlighted (Gaugler, 2005; Majerovitz et al., 2009; Roberts and Ishler, 2017). Also, they want a good relationship with the staff who provide care to their relative with dementia (Ryan and Mckenna, 2015; Hoek et al., 2021; Reid and Chappell, 2017). Some assumptions, for example that family caregivers cannot complete a pain assessment tool because it would be too burdensome or difficult for them, need to be challenged. We found that family caregivers are willing and able to do more than professionals initially thought. They can make valuable contribution to pain observation, assessment and management, as a recent study confirms (Riffin et al., 2023). Nevertheless, it is also clear that involving family caregivers in an intervention continues to be complex (Puurveen et al., 2018; Bauer et al., 2014; Hertzberg et al., 2003). Despite having different perspectives and expectations, the family caregivers and professionals in our study showed a willingness to listen and empathize with the other. There were no obstacles to reaching a consensus to adopt all ideas for the STA OP! *with family*, as professionals recognized the different needs of family caregivers.

Finally, the idea that all care is taken over by nursing home staff after admission of the resident to the nursing home hampers family involvement in the care of their relative. This assumption should be abandoned and there should be more focus on collaboration with family caregivers. This requires a change in culture, especially regarding staff-family collaboration and expectations regarding care. Interventions to increase family involvement are frequently focused on staff-family interaction, and less frequently on the relationship between family members, and between family and resident. These interventions between staff and family are also predominantly focused on staff and few interventions premise an equal relationship between family and staff. Thus, the differences between staff and family persist and the relationship cannot become an equal partnership, where the family has optimal opportunity to play an active role, be in charge and influence the care of their family member. Moreover, the effect of interventions to improve family involvement is still unclear (Backhaus et al., 2020).

Despite the willingness of all involved to contribute to a more family-centered STA OP! method, there are still barriers for professionals and family caregivers to overcome. As other research shows, professionals recognizing family caregivers as partners in care rather than just visitors (Puurveen et al., 2018; Hertzberg et al., 2003), and an environment where professionals and family feel comfortable discussing each other's responsibilities in the resident's care, are unfortunately not yet common in long-term care (Hertzberg et al., 2003; Baumbusch and Phinney, 2014; Bauer et al., 2014). Professionals are less focused on the feelings of uncertainty and grief that family caregivers struggle with. Professionals letting go of certain care routines can help overcome these limitations (Baumbusch and Phinney, 2014; Majerovitz et al., 2009).

Including family caregivers prior to implementation of an intervention is recommended (Tasseron-Dries et al., 2021; Reid and Chappell, 2017; Tan et al., 2022; Baumbusch and Phinney, 2014). This facilitates professionals and family caregivers adopting the intervention and can be the first step toward successful implementation. This study confirms that by involving family caregivers at an early stage, interventions can be tailored to the family caregivers' needs, potentially empowering them to be more actively involved in the care of their relative. The next step is to implement the STA OP! *with family* method in the nursing homes and to examine its effect on residents, professionals and family caregivers. The implementation includes training for professionals on using the adapted manual with information on communication and collaboration with family caregivers, the role of family, and ideas for involving family caregivers in the various steps of the intervention. STA OP! *with family* also requires a slightly modified implementation strategy compared to STA OP! because family caregivers must be involved as an important stakeholder and be informed about their role prior to the implementation. Patient and public involvement is currently recommended, even for an intervention intended for professional (Health Research Authority, 2020).

Finally, ideas on proper communication with family caregivers about the method and their role in the STA OP! *with family* method and about the design of psychoeducation for family caregivers were not discussed in the advisory groups. These findings emphasize that future research should further explore using the practical ideas in the STA OP! *with family* intervention in daily practice. Other studies also state that it is important to take into account the different needs family have in being involved in the care for their relative and the various factors influencing their involvement (Wittenberg et al., 2024a,b). Adopting a family centered approach, with an active and important role for them and making a personal plan that fit their needs can help stimulate their involvement in interventions (Hayward et al., 2022; Tasseron-Dries et al., 2021, 2023). Staff should invest in getting to know the family and their circumstances to create an understanding of what family can and want to do in the care for their relative (Wittenberg et al., 2024b). Future interventions need to focus especially an partnership between family and staff with mutual exchange to enable person-centered care for residents. Further efficacy research is required because little is known about the effect of promising interventions to promote family inclusion in the nursing homes (Backhaus et al., 2020).

Strengths of this study include the involvement of all stakeholders (e.g., family and professional caregivers from all disciplines) in the development of the STA OP! *with family*. Moreover, the formation of the advisory groups, in which professionals and family caregivers participated together, led to discussions and initiated ideas for engaging family caregivers in challenging behavior and pain from both perspectives. Finally, using elements of a framework such as that of Walshe et al. (2019), might promote interprofessional collaboration, which has proven positive outcomes on residents' and professional caregivers' satisfaction with care provided (Reid and Chappell, 2017; Nazir et al., 2013; Fewster-Thuente and Velsor-Friedrich, 2008), and on quality assurance (Kirk et al., 2016; Damschroder et al., 2022).

A limitation is the relatively low number of participants per NGT meeting. Another limitation concerns the absence of family caregivers during the NGT meetings who, although not actively involved in STA OP!, were familiar with the method. This may have resulted in a discussion of the ideas from the professional perspective only. For future studies to enhance family involvement, a more lenient approach to the inclusion criteria of the NGT may be recommended, for example, by also inviting families from organizations that have used the original STA OP! method. Another option would be to collaborate more closely with the client council. This might have led to more family participation in the study. Furthermore, the situation and therefore feasibility of ideas to involve family caregivers in the STA OP! *with family* can differ between nursing homes. Another limitation is that most participants were employed by the same organization. Having participants from other organizations may result is slightly different feasibility ratings. Furthermore, family caregivers may have been extra motivated to participate in the advisory groups, causing possible bias. Also, the advisory groups might have developed feasible ideas without the valuable advice of the experts. In retrospect, the experts' recommendations were more confirmation of what the advisory groups had already assessed as feasible. The ideas are widely applicable and adaptable, making them suitable for any nursing home.

## Conclusion

Family caregivers and professionals collaborating in co-creation clarifies the role family can have in a method for managing challenging behavior and pain. Participants who were naïve to the intervention provided useful and feasible ideas for involving family in the STA OP! method. Further research is needed to examine the impact of STA OP! *with family* on resident, family caregivers, and healthcare professionals. This would include a process evaluation after implementation of the STA OP! *with family* focused on the question what has worked well for whom and in which situation.

## Data Availability

The data supporting the findings of this study and material for the training are available from the corresponding author upon request. The data are not available due to potentially identifying information that could comprise the privacy of research participants.

## Footnotes

^1^ https://osf.io/5ghnx
