# Supplementary material for: Modifying a multidisciplinary method to address challenging behavior in nursing home residents with dementia to involve family caregivers
Source: Front Dement. 2024 Sep 30;3:1444815. doi: 10.3389/frdem.2024.1444815 (PMC11471609; doi:10.3389/frdem.2024.1444815)
Supplement: Supplementary file 1 [file Data_Sheet_1.zip › Supplementary material A.DOCX]

Supplement A: Ideas that received more than 3 votes based on the individual top-5 best ideas of the participants in two advisory group meetings.

**Modifying a multidisciplinary method to address challenging behavior in nursing home residents with dementia to involve family caregivers**

Petra E. M. Tasseron-Dries, MSc^,^ Hanneke J. A. Smaling, PhD Jenny T. van der Steen, PhD^,^ Prof. Wilco P. Achterberg, MD, PhD

| Ideas collected in two advisory groups | Votes* |
| --- | --- |
| Record the resident's life history, but not immediately on admission as that can already be a stressful moment when the family caregiver receives a lot of information. Use a clear diagram, for example a mind map, so that all healthcare professionals at a single glance have an idea of who the resident is before they start providing care. | 12 |
| Dealing with challenging behavior by healthcare professionals and family caregiver:  - discuss behavior (in personal conversations between healthcare professional and family caregiver or in regular meetings such as the multidisciplinary meeting).  - if desired, healthcare professionals explain to the family caregiver how to deal with challenging behavior and how the family caregiver can influence it. | 10 |
| Knowledge transfer about dementia and challenging behavior (psychoeducation) for family caregivers, for example, by organizing family evenings, but also through informal contact between family caregivers and healthcare professionals. | 10 |
| Combine the multidisciplinary meeting and the care plan meeting with family caregiver, allowing the latter to be recognized as a member of the multidisciplinary team. | 9 |
| Also get to know the family caregiver by asking about the relationship between the family caregiver and the resident, what their life was like before admission to the nursing home, and the family caregiver's needs and possibilities regarding being involved. A formal questionnaire on the relationship, and the degree of burden of the family caregiver in caring for the resident can be helpful. | 9 |
| Good contact with the immediate family, including the family caregiver's and resident's network so several relatives can be involved for the challenging behavior, not just the first contact. | 7 |
| Ask family caregivers to record a message (audio or voice/video) to use later when the resident needs reassurance. | 6 |
| Family caregiver and healthcare professionals devise appropriate activities for family to do together with the resident or for the resident (e.g., go for a walk, tidy up closet, eat together). | 6 |
| Report not only on medical aspects, but also on well-being, emotions, on mood, and how the resident is ‘really’ doing. Not just things like: She went to the toilet. But also: She felt very happy, smiled all the time and sang along with ..., etc.). | 6 |
| Together with the family caregiver, the healthcare professional maps the resident's life course and history. Making a mind map or collage can be helpful. | 6 |
| Healthcare professional and family caregiver assist resident with washing and dressing (e.g., family caregiver can help, observe, and give advice). | 5 |
| The family caregiver completes a pain score list about their relative. | 5 |
| Facilitate the resident and family caregiver having meals together (on the ward/room). | 4 |
| Organize an information meeting for family caregiver on the course of dementia (psychoeducation). | 4 |

* Participants brainstormed about concrete ideas to involve family caregivers in the STA OP! method. After each idea was discussed, including possible barriers and solutions to those barriers, all participants selected their personal top-5 best ideas to involve family caregivers.
